# Interactions between ZnO Nanoparticles and Polyphenols Affect Biological Responses

**DOI:** 10.3390/nano12193337

**Published:** 2022-09-25

**Authors:** Su-Bin Kim, Na-Kyung Yoo, Soo-Jin Choi

**Affiliations:** Division of Applied Food System, Major of Food Science & Technology, Seoul Women’s University, Seoul 01797, Korea

**Keywords:** zinc oxide nanoparticles, quercetin, rutin, interactions, biological responses, characteristics, surface chemistry

## Abstract

Zinc oxide (ZnO) nanoparticles (NPs) are used as a food additive Zn supplement due to the role of Zn in biological functions. They are directly added to complex processed foods or Zn-fortified functional foods. Hence, the interactions between ZnO NPs and nutritional or functional components can occur. In this study, the effects of ZnO NP interactions with two polyphenols (quercetin and rutin) on cytotoxicity, antioxidant activity, ex vivo intestinal absorption, and solubility were evaluated. Moreover, the characterization on the interactions was carried out by analyzing crystallinity, surface chemical bonding, chemical composition, and surface chemistry. The results demonstrate that the interactions caused higher cytotoxicity, ex vivo intestinal transport, and solubility of ZnO NPs than pristine ZnO NPs but did not affect antioxidant activity nor intestinal absorption of the polyphenols. The interaction effects were more evident by ZnO NPs interacted with quercetin than with rutin. The crystallinity of ZnO NPs was not influenced, but the degree of exposure of the chemical bondings, elemental compositions, and chemical group intensities on the surface of ZnO NPs, quercetin, or rutin were quenched or decreased to some extent by the interactions, especially by ZnO NPs interacted with quercetin. It is, therefore, concluded that the interactions affect chemical characteristics and surface chemical sates of ZnO NPs, quercetin, or rutin, which can cause high cytotoxicity, intestinal absorption, and solubility of ZnO NPs. Further study is required to elucidate the mechanism of action of the interactions.

## 1. Introduction

Food additive zinc oxide (ZnO) is utilized as a Zn supplement because an essential trace element Zn plays a role in diverse cellular functions, such as enzymatic process, cell-mediated immunity, transcription factor, cell growth, wound healing, and organ development [1,2,3,4]. The Zn salts including ZnO have been traditionally applied to facilitate wound healing [5]. ZnO is also widely applied to food packaging to preserve the quality and to extend shelf-life of foods due to its antimicrobial action [6,7,8,9]. Moreover, ZnO is listed as a “generally recognized as safe (GRAS)” material by Food and Drug Administration in the United States (21CFR182.8991) [10]. ZnO nanoparticles (NPs) were reported to have more efficient antimicrobial and biological activities than micro-sized particles related to small size (1–100 nm) and large surface-to-volume ratio [11,12]. Hence, it is probable that the toxicity and biological responses of ZnO NPs are different from bulk-sized particles. High Zn intakes can also cause undesirable effects, including flu-like symptoms, vomiting, diarrhea, and stomach ache [13,14].

When ZnO is applied as a Zn supplement, it is directly added to complex processed foods where diverse food components, such as carbohydrates, proteins, lipids, and other minor nutrients, are present. Furthermore, it is often added in Zn-fortified functional foods, and thus, the interactions between ZnO NPs and nutritional or functional components can occur to some extent. The interaction may alter the potential toxicity of ZnO NPs and functionality of nutritional or functional components. Indeed, Cao et al. reported that the cytotoxicity of ZnO NPs increased in the presence of palmitic acid associated with increased generation of mitochondrial reactive oxygen species (ROS) in Caco-2 cells, whereas the ROS generation was not affected by the presence of free fatty acids [15]. Wang et al. showed that a functional food additive, caseinophosphopeptides (CPP), protects human gastric epithelium cells (GES-1) from oxidative stress caused by ZnO NPs [16]. On the other hand, a synergistic toxicity of ZnO NPs and vitamin C was demonstrated in both GES-1 cells and a mouse model [17]. Different order of addition of CPP, vitamin C, and ZnO NPs was also reported to affect cytotoxicity and intestinal absorption of Zn ions in an everted gut sac model [18]. However, the combined effect of ZnO NPs and food components on the toxicity and bio-responses has not been extensively explored, although various food matrices, additives, functional compounds, and nutrients are present in processed foods.

Quercetin and rutin are polyphenolic flavonoid compounds abundantly present in various foods and plants, and they act as natural antioxidants [19,20]. They possess free radical scavenging activity, and thereby play vital roles in protection against oxidative stress, inflammation, certain forms of cancer, pulmonary and cardiovascular diseases, and aging [20,21,22]. In this study, we hypothesized that the interactions could alter the potential toxicity, physico-chemical characteristics, and bio-responses of ZnO NPs or functional compounds. The effects of the interactions between ZnO NPs and representative two polyphenols (quercetin and rutin) on cytotoxicity, antioxidant activity, dissolution property, and ex vivo intestinal transport amount were investigated. Moreover, the characterization of the interactions was further carried out by analyzing particle size, crystalline phase, chemical sates, and surface chemistry.

## 2. Materials and Methods

### 2.1. Materials and Preparation

ZnO NPs (<100 nm), quercetin, 2,2′azinobis-(3-ethyl-benzothiazoline-6-sulfonic acid) (ABTS), potassium persulfate (K_2_SO_4_), calcium chloride dihydrate (CaCl_2_·2H_2_O), potassium thiocyanate (KSCN), sodium bicarbonate (NaHCO_3_), urea, α-amylase, uric acid, mucin, D-(+)-glucose, glucuronic acid, glucosamine hydrochloride, bovine serum albumin (BSA), pepsin, lipase, bile, monosodium phosphate (NaH_2_PO_4_), and Zn standard solution were purchased from Sigma-Aldrich (St. Louis, MO, USA). Rutin trihydrate and 2,2-diphenyl-2-picrylhydrazyl (DPPH) were provided by Alfa Aesar (Ward Hill, MA, USA). L-ascorbic acid, nitric acid (HNO_3_), hydrogen peroxide (H_2_O_2_), sodium chloride (NaCl), potassium chloride (KCl), potassium dihydrogen phosphate (KH_2_PO_4_), calcium chloride (CaCl_2_), acetic acid, methanol, acetonitrile, ethyl alcohol, and hydrochloride (HCl) were obtained from Samchun Pure Chemical Co., Ltd. (Pyeongtaek, Korea). Sodium sulfate (Na_2_SO_4_), sodium dihydrogen phosphate dihydrate (NaH_2_PO_4_·2H_2_O), and magnesium chloride (MgCl_2_) were purchased from Junsei Chemical Co., Ltd. (Tokyo, Japan). 3-(4,5-dimethyl thiazol-2-yl)-2,5-diphenyltetrazolium bromide (MTT) and 2′,7′-dichlorofluoroescein diacetate (H_2_DCFDA) were provided by Duchefa Biochemie (Haarlem, Netherlands) and Molecular Probes, Inc. (Eugene, OR, USA), respectively. Minimum essential medium (MEM), phosphate-buffered saline (PBS), inactivated fetal bovine serum (FBS), penicillin, and streptomycin were obtained from Welgene Inc. (Gyeongsan, Gyeongsangbuk-do, Korea). A CytoTox 96 Nonradioactive Cytotoxicity Assay kit and sodium dodecyl sulfate (SDS) were purchased from Promega (Madison, WI, USA) and Elpis Biotech Inc. (Daejeon, Korea), respectively.

ZnO NPs pre-incubated with polyphenols (quercetin or rutin) by stirring for 30 min were presented as ‘ZnO + polyphenols w pre-incubation’ in Figures and Tables. ZnO NPs mixed only with polyphenols just before sample treatments without pre-incubation were presented as ‘ZnO + polyphenols *w/o* pre-incubation’ in Figures and Tables.

### 2.2. Physico-Chemical Characterization

The constituent particle size and shape of ZnO NPs were examined by field emission scanning electron microscopy (FE-SEM; JSM-7100F, JEOL, Tokyo, Japan). The average particle size and size distribution were measured by randomly selecting more than 100 particles from the SEM image using ImageJ software (version 1.53k, National Institutes of Health, Bethesda, MD, USA). The hydrodynamic diameters and zeta potentials of ZnO NPs were measured by preparing ZnO NPs (100 μg/mL) in distilled water (DW), MEM, or Tyrode’s solution with or without pre-incubation with polyphenols (312.5 μM). The concentrations of ZnO NPs and polyphenols in three consecutive steps of digestion fluid were 1 mg/mL and 3.125 mM, respectively. Then, the hydrodynamic diameters and zeta potentials of ZnO NPs were evaluated by means of dynamic light scattering and electrophoretic light scattering, respectively, using a Zetasizer Nano System (Malvern Instruments, Worcestershire, UK).

For powder X-ray diffraction (XRD), Fourier transformed infrared (FTIR), and X-ray photoelectron spectroscopy (XPS) analysis, ZnO NPs (100 μg/mL) were pre-incubated with quercetin or rutin (312.5 μM) for 30 min and completely dried at room temperature in powdered forms. The powder XRD patterns were analyzed using a diffractometer (SmartLab, Rigaku Co., Tokyo, Japan) with Ni-filtered CuKα radiation (λ = 1.5418 Å, a voltage of 40 kV, a current of 40 mA, a scan range of 5–80° with a step size of 0.02°, and a scanning rate of 3°/min). The FTIR (Spectrum one System, Perkin Elmer, Waltham, MA, USA) analysis was conducted with the standard KBr disk method. Briefly, 100 mg of KBr was pulverized with 1 mg of the samples in a mortar. The KBr pellet was formed by handpress with a 7 mm die set (PIKE technologies, Fitchburg, WI, USA), and the FTIR spectra were recorded in the regions from 400 cm^−1^ to 4000 cm^−1^ with 8 cm^−1^ resolution and 16 replicate scans. The surface chemistry and chemical compositions were evaluated by XPS (K-Alpha XPS, Thermo Fisher Scientific, Waltham, MA, USA) using X-ray source of Al-Kα. The samples were fixed on a sample holder using a conductive carbon tape. Survey spectra were analyzed at 200 eV pass energy and 1.0 eV energy step of the analyzer and obtained from 1350 eV to 0 eV. The high-resolution spectra for Zn 2p, C 1s, and O 1s were measured at 50 eV pass energy and 0.1 eV energy step. The binding energies were corrected for the charge shift by setting the C1s peak at 284.6 eV. The obtained high-resolution spectra were fitted using Igor Pro software (version 9, Wavemetrics, Lake Oswego, OR, USA).

### 2.3. Cytotoxicity

#### 2.3.1. Cell Culture

Human intestinal epithelial Caco-2 cells were purchased from Korean Cell Line Bank (Seoul, Korea), and cultured in MEM containing 10% FBS, 100 units/mL of penicillin, and 100 μg/mL of streptomycin under 5% CO_2_ atmosphere at 37 °C.

#### 2.3.2. Cell Proliferation

The effect of the interactions between ZnO NPs (20 μg/mL) and polyphenols (quercetin and rutin) on cell proliferation was estimated by MTT assay. Cells (1×10^4^ cells/100 μL) were treated with the samples for 24 h in a 96-well plate, and 10 μL of MTT solution was added. After further incubation for 4 h, the cells were treated with 100 μL of solubilization solution (0.01 M HCl + 10% SDS) and incubated overnight. The absorbance was measured at 570 nm using a microplate reader (SpectraMax^®^ M3, Molecular Devices, Sunnyvale, CA, USA). Cells incubated without samples were used as controls.

#### 2.3.3. Lactate Dehydrogenase (LDH) Leakage

The effects of the interactions between ZnO NPs (20 μg/mL) and polyphenols on cell membrane damage and integrity were assessed by measuring intracellular LDH release into extracellular medium using a CytoTox 96 Nonradioactive Cytotoxicity Assay kit. Cells (4 × 10^4^ cells/mL) were incubated with the samples in a 24-well plate for 24 h. Then, the cell culture medium was collected and centrifuged. Aliquots (50 μL) of the supernatants were treated with a substrate solution (50 μL) in the dark at room temperature. After 30 min, a stop solution (50 μL) was added, and the absorbance was measured at 490 nm with a microplate reader (SpectraMax^®^ M3, Molecular Devices). Cells incubated without samples were used as controls.

#### 2.3.4. Reactive Oxygen Species (ROS) Generation

The ROS generated by the interactions between ZnO NPs (20 μg/mL) and polyphenols were monitored with a peroxide-sensitive fluorescent probe, H_2_DCFDA. Cells (1 × 10^4^ cells/100 μL) were incubated with the samples in a 96-well plate for 24 h. The cells were then treated with 20 μM H_2_DCFDA for 30 min at 37 °C in the dark. After washing with PBS, dichlorofluorescein (DCF) fluorescence was immediately measured using a fluorescence microplate reader (SpectraMax^®^ M3, Molecular Devices). Excitation and emission wavelengths were set at 485 nm and 535 nm, respectively. Cells incubated without samples were used as controls.

### 2.4. Radical Scavenging Activity

Antioxidant activity of quercetin or rutin (312.5 μM) in the presence of ZnO NPs (100 μg/mL) was evaluated by radical ABTS and DPPH scavenging assays. The ABTS radicals were generated by mixing 10 mM ABTS and 10 mM K_2_SO_4_ with a ratio of 7.4:2.6 (*v*/*v*) at 37 °C for 24 h in the dark. After dilution with PBS to appropriate concentrations, 150 μL of ABTS radical solution was added to 50 μL of the samples, and the solutions were incubated at room temperature in the dark. After 30 min, absorbance was measured at 734 nm using a microplate reader (SpectraMax^®^ M3, Molecular Devices). Antioxidant activity was expressed as L-ascorbic acid equivalent (AAE mg/100 mL sample).

For the DPPH assay, 100 μL of DPPH solution was added to 100 μL of the samples, and the solutions were incubated at room temperature. After 30 min, the absorbance was measured at 517 nm using a microplate reader (SpectraMax^®^ M3, Molecular Devices). Antioxidant activity was expressed as L-ascorbic acid equivalent (AAE mg/100 mL sample).

### 2.5. Dissolutuion Property of ZnO NPs in Digestion Fluids

The effect of the interactions between ZnO NPs and polyphenols on the solubility of ZnO NPs was evaluated using a simulated three-steps of digestion fluids as described by Peters et al. (Table S1) [23]. All simulated digestion fluids were prepared and pre-heated at 37 °C for 2 h on the day of the experiment. For dissolution experiments in a single digestion system, ZnO NPs (1 mg/mL) interacted with quercetin or rutin (3.125 mM) were dispersed in simulated saliva, gastric fluid, and intestinal fluid, and incubated for 5 min, 2 h, and 2 h, respectively, on a head-over-head rotator at 37 °C. For dissolution experiments in three consecutive steps of digestion system, ZnO NPs (1 mg/mL) interacted with quercetin or rutin (3.125 mM) were incubated in simulated saliva (6 mL) at 37 °C for 5 min. After the simulated gastric fluid (12 mL) was added, the mixtures were incubated at 37 °C for 2 h, followed by further digestion for 2 h at 37 °C after addition of duodenal fluids (12 mL) and bile fluids (6 mL). The digested samples were centrifuged at 16,000× *g* for 15 min, and the concentrations of dissolved Zn from ZnO NPs in the supernatant were quantified using inductively coupled plasma-atomic emission spectroscopy (ICP-AES; JY2000 Ultrace, HORIBA Jobin Yvon, Longjumeau, France).

### 2.6. Quantitative Analysis

Quantitative analyses of ZnO NPs were carried out by digesting the samples with 60% HNO_3_ (10 mL) at ~160 °C. After addition of 34.5% H_2_O_2_ (1 mL), the mixtures were heated until the samples were colorless. The digested samples were diluted with appropriate volumes of distilled and deionized water after evaporation of the solutions, and the total Zn concentrations were measured by ICP-AES (JY2000 Ultrace, HORIBA Jobin Yvon) with a radiofrequency power of 1000 W and a plasma gas flow of 12 L/min.

The concentrations of quercetin and rutin were determined by high-performance liquid chromatography (HPLC; Agilent 1100 series, Agilent Technologies, Santa Clara, CA, USA) equipped with a variable wavelength detector. Supelcosil^TM^ LC-18 column (250 mm × 4.6 mm i.d., 5 μm, Supelco Inc., Bellefonte, PA, USA) was used as stationary phase and the mobile phase was methanol: acetonitrile: water (1:2:7, *v*/*v*/*v*) containing 2.5% acetic acid. The samples were filtered using a 0.45 μm of syringe filter (Advantec, Techigi, Japan) and analyzed with the detection wavelength of 350 nm, flow rate of 1 mL/min, and injection sample volume of 20 μL.

### 2.7. Intestinal Absorption Using an Everted Small Intestinal Sacs

Seven-week-old male Sprague-Dawley rats were purchased from Koatech Co. (Pyeongtaek, Gyeonggi-do, Korea). The rats were housed in laboratory plastic animal cages in a ventilated clean rack maintained at 20 ± 2 °C and 60 ± 10% relative humidity with a 12 h light–dark cycle. The rats were given access to a standard laboratory complete diet and water ad libitum. The animals were acclimatized for 7 d before experiments. Animal experiments were performed in accordance with guidelines for the Institutional Animal Care and Use Committee (IACUC) of the Seoul Women’s University. Protocols used in this study were approved by the Seoul Women’s University (SWU IACUC-2020A-1).

Ex vivo everted small intestinal sacs were prepared as previously described by Gu et al. [18]. Briefly, two male rats were euthanized by CO_2_ after being fasted overnight with available water. The small intestines were obtained and washed three times with Tyrode’s solution (containing 0.8 g of NaCl, 0.02 g of KCl, 0.02 g of CaCl_2_, 0.01 g of MgCl_2_, 0.1 g of NaHCO_3_, 0.005 g of NaH_2_PO_4_, and 0.1 g of glucose in 100 mL of DW). Then, the small intestines were cut into sections (5 cm in length) and everted using a puncture needle (0.8 mm in diameter). After one end was clamped, the everted intestinal sacs were filled with 200 μL of Tyrode’s solution and tied using silk braided sutures. The intestinal sac was incubated with 3 mL of the samples (100 μg/mL ZnO NPs, 312.5 μM quercetin or rutin) in a 6-well plate in a humidified 5% CO_2_ atmosphere at 37 °C. After 2 h, the solutions inside the intestinal sacs were collected and the concentrations of absorbed ZnO NPs and polyphenols were analyzed by ICP-AES (JY2000 Ultrace, HORIBA Jobin Yvon) and HPLC (Agilent 1100 series, Agilent Technologies), respectively, as described in “2.6. Quantitative Analysis”.

### 2.8. Statistical Analysis

All data were presented as means ± standard deviations. Statistical significance of intergroup differences was determined by performing a one-way analysis of variance with Tukey’s Test in SAS (version 9.4, SAS Institute Inc., NC, USA) at *p* values < 0.05.

## 3. Results and Discussion

### 3.1. Particle Size, Size Distribution, and Hydrodynamic Diameters of ZnO NPs

The constituent particle size, shape, and size distribution of ZnO NPs were analyzed by SEM. Figure S1 shows that ZnO NPs had an average particle size of 73.2 ± 15.7 nm with irregular round or oval shapes. The hydrodynamic diameters and zeta potentials of ZnO NPs in DW were determined to be 346 ± 9 nm and 18.5 mV, respectively (Table 1), suggesting the formation of agglomerates or aggregates in aqueous solution. The hydrodynamic diameters of ZnO NPs reduced in the presence of rutin both without and with pre-incubation, whereas quercetin with pre-incubation only reduced their hydrodynamic diameters in DW. The same tendency was found in cell culture medium MEM.

On the other hand, remarkably reduced hydrodynamic diameters of ZnO NPs were found in the presence of quercetin or rutin only after pre-incubation in Tyrode’s solution (isotonic with intestinal fluid) and consecutive three-steps of digestion fluids (Table 1). It is worth noting that ZnO NPs pre-incubated with quercetin were less aggregated than ZnO NPs with rutin in Tyrode’s and digestion solutions where ex vivo intestinal absorption and solubility were evaluated. Positive zeta potentials of ZnO NPs changed to negative charges in the presence of quercetin or rutin in DW, suggesting the interactions between ZnO NPs and the polyphenols. Changes in zeta potentials of ZnO NPs by the interactions were not remarkable in MEM, Tyrode’s solution, and digestion fluids, likely due to the presence of various components in these biological solutions. Dramatically increased hydrodynamic diameters of ZnO NPs in digestion fluids were attributed to the formation of Zn aggregates with carbonate or phosphate anions present in the intestine, as presented in previous reports [24,25]. All the results suggest that the interactions reduced hydrodynamic diameters of ZnO NPs, and especially, ZnO NPs pre-incubated with quercetin could significantly reduce the hydrodynamic diameters in simulated biological fluids including Tyrode’s and digestion solutions.

### 3.2. Interaction Effect on Cytotoxicity

**Figure 1:**
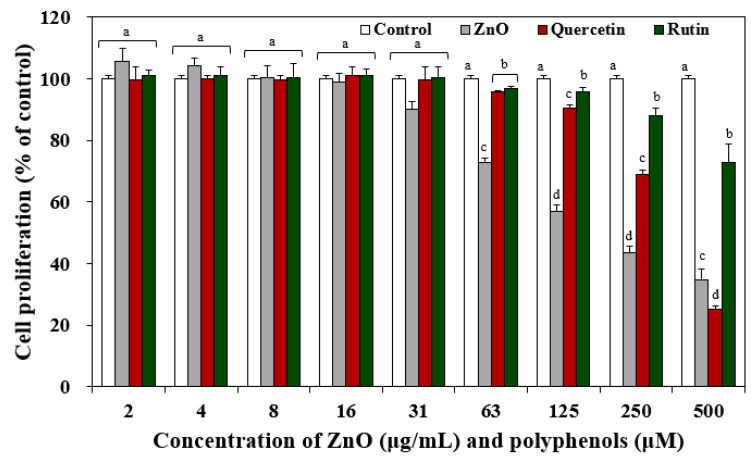
Effect of pristine ZnO NPs and two polyphenols (quercetin and rutin) on cell proliferation of Caco-2 cells after 24 h. Different lower-case letters (a,b,c,d) indicate significant differences between quercetin and rutin (*p* < 0.05).

The effect of pristine ZnO NPs, quercetin only, and rutin only on cell proliferation of human intestinal Caco-2 cells was evaluated by MTT assay. The results showed that cell proliferation was significantly inhibited by ZnO NPs and two polyphenols above 63 μg/mL and above 63 μM (Figure 1), respectively. When the cytotoxicity of two polyphenols was compared, higher cytotoxicity of quercetin than rutin was found (Figure 1).

**Figure 2:**
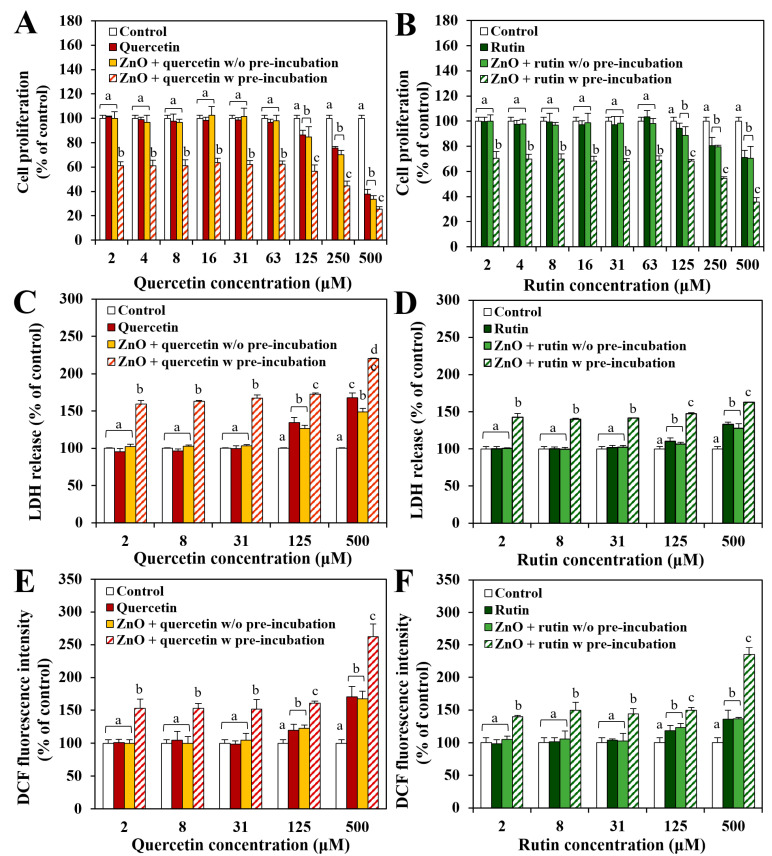
Effect of the interactions between ZnO NPs and two polyphenols (quercetin and rutin) with (*w*) or without (*w/o*) pre-incubation on (**A**,**B**) cell proliferation, (**C**,**D**) lactate dehydrogenase (LDH) release, and (**E**,**F**) reactive oxygen species (ROS) generation in Caco-2 cells after 24 h. Different lower-case letters (a,b,c,d) indicate significant differences among untreated control, quercetin or rutin only, ZnO in the presence of quercetin or rutin without (*w/o*) or with (*w*) pre-incubation (*p* < 0.05). Abbreviations: LDH, lactate dehydrogenase; DCF, dichlorofluorescein.

The effect of the interactions between ZnO NPs and quercetin or rutin on cytotoxicity was investigated by exposing the cells to ZnO NPs at 20 μg/mL, where no cell proliferation inhibition was found (Figure 1), pre-incubated with polyphenols for 30 min, or just in the presence of polyphenols without pre-incubation (Figure 2). Figure 2A,B demonstrate that ZnO NPs in the presence of polyphenols without pre-incubation inhibited cell proliferation in a similar manner to quercetin or rutin only, whereas ZnO NPs pre-incubated with polyphenols caused significantly higher cell proliferation inhibition compared with ZnO NPs in the presence of polyphenols without pre-incubation or polyphenols only. The same tendency was found in terms of LDH release (Figure 2C,D) and ROS generation (Figure 2E,F). Significantly reduced hydrodynamic diameters of ZnO NPs pre-incubated with quercetin or rutin in MEM (Table 1) may contribute to causing high cytotoxicity [26,27,28].

When the interaction effect of two polyphenols was compared, slightly higher cytotoxicity of ZnO NPs pre-incubated with quercetin than with rutin was found in all cases. The higher toxicity of the former than the latter is likely to be associated with high toxicity of quercetin itself (Figure 1). It is worth noting that the hydrodynamic diameters of the former were significantly larger than the latter in MEM (Table 1), and pristine ZnO NPs at 20 μg/mL did not inhibit cell proliferation (Figure 1). Hence, small particle size is not the only factor affecting cytotoxicity. The results indicate that ZnO NPs pre-incubated with quercetin or rutin can exhibit higher cytotoxicity than pristine ZnO NPs, quercetin only, or rutin only, suggesting synergistic effect of the interactions between ZnO NPs and polyphenols on the cytotoxicity.

### 3.3. Interaction Effect on Antioxidant Activity of Polyphenols

**Figure 3:**
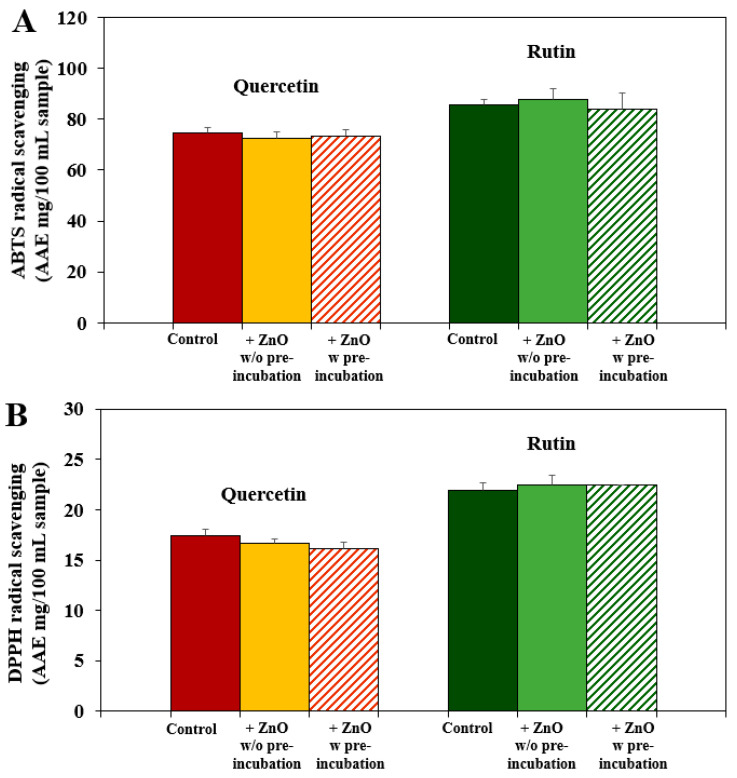
(**A**) ABTS and (**B**) DPPH radical scavenging activities of quercetin or rutin (control) in the presence of ZnO NPs with (*w*) or without (*w/o*) pre-incubation. No significant differences were found among control (quercetin or rutin), quercetin, or rutin in the presence of ZnO without (*w/o*) or with (*w*) pre-incubation (*p* > 0.05).

Antioxidant activity of quercetin or rutin in the presence of ZnO NPs with or without pre-incubation was evaluated by ATBS and DPPH radical scavenging assays. Figure 3 shows that the ABTS (Figure 3A) and DPPH (Figure 3B) scavenging activities of quercetin and rutin were not affected by the interactions with ZnO NPs even after pre-incubation. These results suggest that the interactions between ZnO NPs and quercetin or rutin do not affect radical scavenging activity of the polyphenols. It seems that the interactions have more impact on cytotoxicity of ZnO NPs than antioxidant function of polyphenols.

### 3.4. Interaction Effect on Ex Vivo Intestinal Absorption

**Figure 4:**
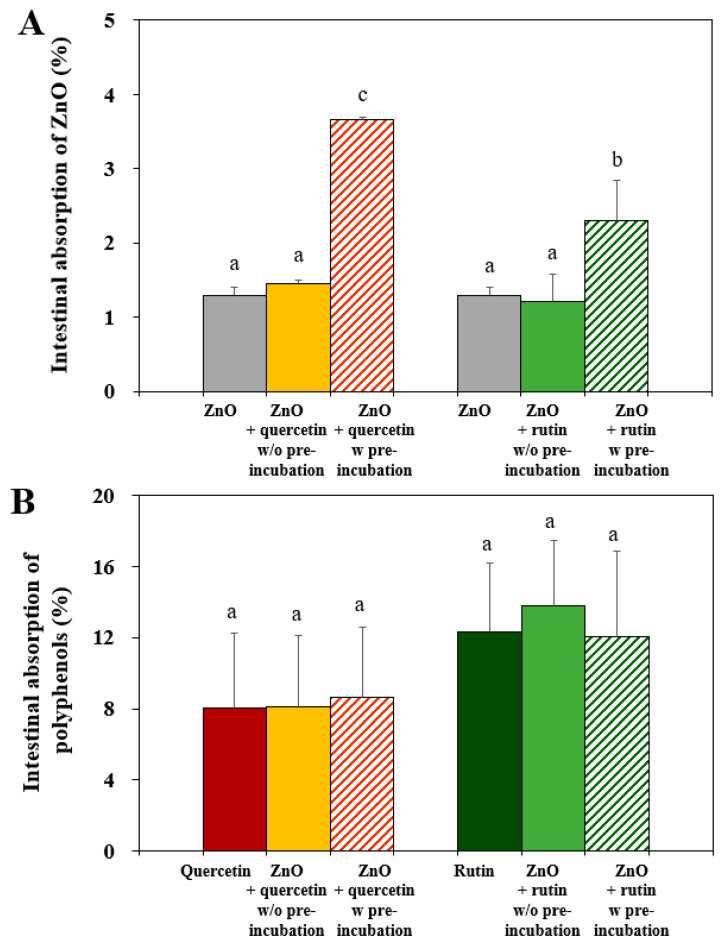
Ex vivo intestinal absorption of (**A**) ZnO NPs and (**B**) quercetin or rutin by the interactions between ZnO NPs and quercetin or rutin using an everted rat small intestinal sac. Different lower-case letters (a,b,c) indicate significant differences among control (ZnO, quercetin, or rutin only) and ZnO in the presence of quercetin or rutin without (*w/o*) or with (*w*) pre-incubation (*p* < 0.05).

The intestinal transport amounts of ZnO NPs, quercetin, and rutin were evaluated using an everted rat small intestinal sac model [18]. Cellular uptake could not be evaluated due to high cytotoxicity caused by the interactions (Figure 2). The results demonstrate that the intestinal transport amounts of ZnO NPs pre-incubated with quercetin or rutin significantly increased, whereas the transport levels of ZnO NPs in the presence of quercetin or rutin without pre-incubation were statistically the same as that of pristine ZnO NPs (Figure 4A). When the interactions between ZnO NPs and two polyphenols were compared, ZnO NPs pre-incubated with quercetin more significantly increased the intestinal absorption than ZnO NPs with rutin. The small hydrodynamic diameters of the former compared with the latter in an isotonic intestinal fluid, Tyrode’s solution, (Table 1) can contribute to high intestinal transport [29,30]

On the other hand, no effect of the interactions on the intestinal absorptions of quercetin or rutin was found regardless of pre-incubation (Figure 4B). The results imply that the interactions between ZnO NPs and quercetin or rutin can enhance the intestinal oral absorption of ZnO NPs, but not that of the polyphenols. Based on the interaction effects on cytotoxicity (Figure 2), radical scavenging activity (Figure 3), and intestinal transport (Figure 4), it is highly likely that the interactions affect the biological responses of ZnO NPs, but not those of two polyphenols. Increased intestinal absorption of ZnO NPs by the interactions could cause potential high toxicity, needing further investigation on the in vivo interaction effects.

### 3.5. Interaction Effect on Dissolution Property of ZnO NPs

**Figure 5:**
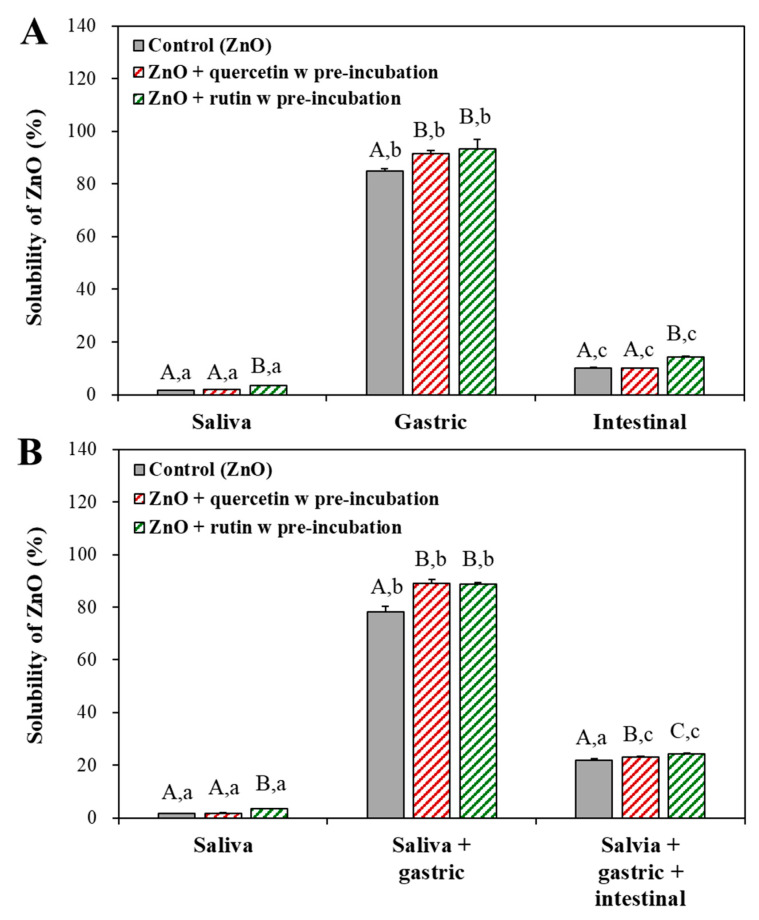
Dissolution properties of ZnO NPs pre-incubated with quercetin or rutin in (**A**) a single digestion and (**B**) consecutive digestion systems. Different upper-case letters (A,B,C) indicate significant differences among control (ZnO) and ZnO pre-incubated with quercetin or rutin (*p* < 0.05). Different lower-case letters (a,b,c) indicate significant differences among digestion fluid systems (*p* < 0.05).

The dissolution property of ZnO NPs interacted with quercetin or rutin was evaluated because ZnO NPs are well known to dissolve under biological and acidic environments to some extent [31,32,33]. The interaction effects were further investigated only after pre-incubation as ZnO interactions without pre-incubation had no different cytotoxicity and ex vivo intestinal absorption, compared with pristine ZnO NPs, quercetin only, or rutin only (Figure 2 and Figure 4). Figure 5 shows that the solubility of ZnO NPs increased in order of saliva (~1.6–3.5%) < intestinal (~10–14%) < gastric (~84–93%) fluids in a single digestion fluid model (Figure 5A), and the same tendency was found in consecutive digestion systems (Figure 5B), as previously reported [24]. Slightly but significantly increased solubility of ZnO NPs was found by the interactions. In particular, ZnO interaction with rutin significantly increased the solubility in neutral pH saliva and intestinal fluids compared with ZnO interaction with quercetin, which was not found in low pH gastric fluid. It is likely that the solubility of ZnO NPs under low pH condition was too high to observe slight differences in the solubility caused by the interactions. It is interesting to note that ZnO NPs pre-incubated with rutin had larger hydrodynamic diameters than ZnO NPs with quercetin in digestion fluids (Table 1). Small-sized NPs were often reported to have high solubility compared with larger-sized particles [34,35,36], which was not the case in the present study. Hence, particle size is not a critical factor affecting the solubility of ZnO NPs interacted with polyphenols. Although the dissolution levels of ZnO NPs differed from digestion fluids tested, all the results demonstrated that the interactions between ZnO NPs and quercetin or rutin can enhance the dissolution property of ZnO NPs in the gastrointestinal fluid. Increased solubility of ZnO NPs by the interactions also implies that the surface characteristics of ZnO NPs may change or be modified by the interactions, requiring further investigation on physico-chemical properties and surface chemistry of ZnO NPs interacted with quercetin or rutin.

### 3.6. Interaction Effect on Physico-Chemical Properties

**Figure 6:**
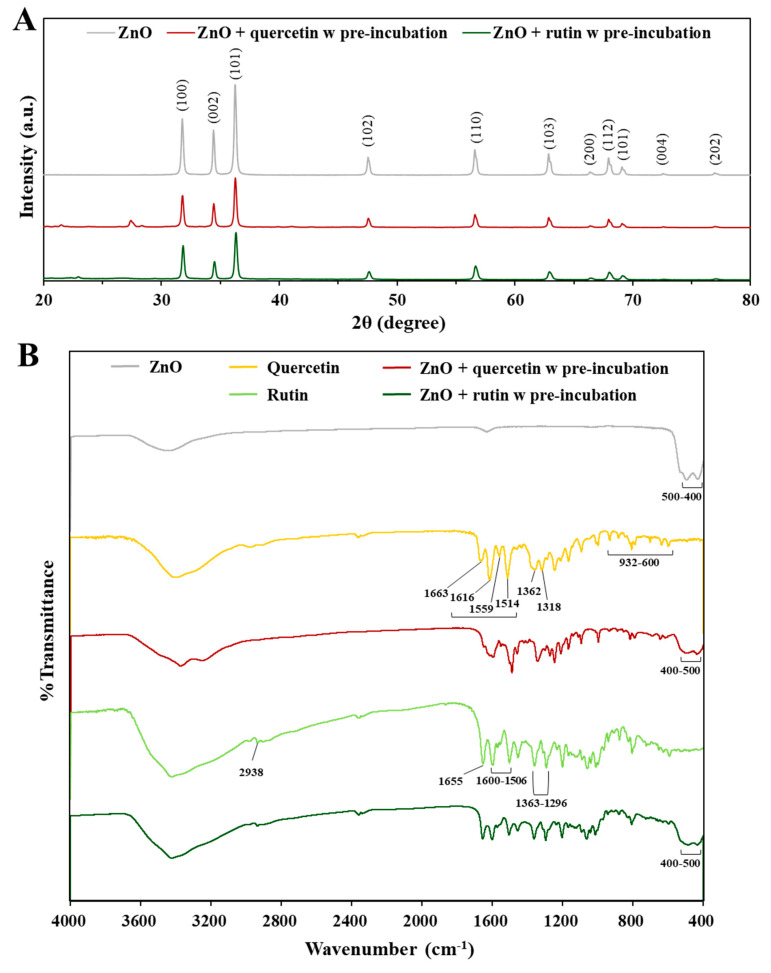
(**A**) X-ray diffraction (XRD) patterns of pristine ZnO NPs and ZnO NPs pre-incubated with quercetin or rutin. (**B**) Fourier transform infrared spectroscopic (FTIR) spectra of pristine ZnO NPs, quercetin or rutin only, and ZnO NPs pre-incubated with quercetin or rutin.

The crystallinity of ZnO NPs was analyzed by XRD in the absence and presence of two polyphenols. The XRD patterns revealed that pristine ZnO had typical wurtzite structure and the crystalline structure was not affected by interactions with quercetin or rutin (Figure 6A) [37]. A peak at 25–30 degrees appeared after pre-incubation with quercetin, which was attributed to the incorporation of water molecules within the lattices, forming hydrogen bonds between quercetin and water [38,39]. A slight peak at 20–25 degrees after pre-incubation with rutin was related to three intermolecular hydrogen bonds between rutin molecules, which is a characteristic of the crystal packing [40]. Hence, it seems that the interactions were not that strong to affect the crystalline phase of ZnO NPs.

FTIR spectroscopic analysis was performed in the range from 400 to 4000 cm^−1^ to investigate the changes in chemical bonding intensity caused by the interactions. The FTIR spectrum of ZnO shows that the characteristic stretching mode of Zn–O bond were found at 400–500 cm^−1^, whereas broad peaks at 3448 and 1637 cm^−1^ correspond to O–H stretching and O–H bending of hydroxyl residue, respectively, due to the presence of atmospheric moisture (Figure 6B) [41]. Pure quercetin shows the typical FTIR spectrum [42]; O–H stretching and O–H bending of the phenolic group were detected at 3400–3220 cm^−1^ and 1362 cm^−1^, respectively. The C=O aryl ketonic stretch was found at 1663 cm^−1^ and the C=C aromatic ring stretch bands were evident at 1616, 1559, 1514 cm^−1^. The in-plane bending band of C–H in aromatic hydrocarbon at 1318 cm^−1^ and out-of-plane bending bands of C–H at 932, 808, 703, and 600 (932–600) cm^−1^ were found. Absorption bands at 1246, 1212, and 1167 cm^−1^ belong to the C–O stretching in the aryl ether ring, the C–O stretching in phenol, and the C–CO–C stretching and bending in ketone, respectively. However, transmittance (%) of most characteristic bands from quercetin remarkably decreased or quenched by interaction with ZnO NPs, indicating the interactions between ZnO NPs and quercetin. Slightly quenched typical Zn-O bond was also found at 400–500 cm^−1^ in ZnO pre-incubated with quercetin, supporting the interactions. On the other hand, the FTIR spectrum of rutin hydrate shows the O–H stretching bond at 3400–3220 cm^−1^ (Figure 6B) [43]. The valence vibrations of the C–H stretching bonds in the CH and CH_2_ groups were noticed at 2938 cm^−1^. The bands at 1655, 1600–1506, and 1363–1296 cm^−1^ were attributed to O–H, C=C, and C–O bands, respectively. The C–O–C bonds were detected at 1204, 1042, and 1014 cm^−1^. After pre-incubation with ZnO NPs, the valence variations of typical bands from rutin slightly decreased but were not completely quenched. It is worth noting that Zn–O band from ZnO at 400–500 cm^−1^ was also slightly quenched by pre-incubation with rutin.

The results clearly indicate that the interactions between ZnO NPs and the polyphenols surely occurred, and the interaction effect on the surface characteristics was more evident by ZnO interaction with quercetin than with rutin, suggesting stronger interaction of the former than the latter. These results also suggest that the interactions can quench the degree of direct exposure of the surface chemical bondings from ZnO, quercetin, or rutin to some extent, which may lead to different biological responses. Relatively strong ZnO interaction with quercetin may lead to causing high cytotoxicity (Figure 2) and ex vivo intestinal transports (Figure 4) compared with ZnO interaction with rutin. On the other hand, slightly high dissolution of ZnO NPs interacted with rutin may be related to relatively week interaction compared with the interaction between ZnO and quercetin. It should be noted that the hydrodynamic diameters of the former were larger than the latter in digestion fluids (Table 1). This result also suggests that the change in particle size by the interactions is not the only factor affecting biological responses and solubility, but the interaction effect on surface characteristics must be also considered.

### 3.7. Interaction Effect on Chemical Composition and Surface Chemistry

**Figure 7:**
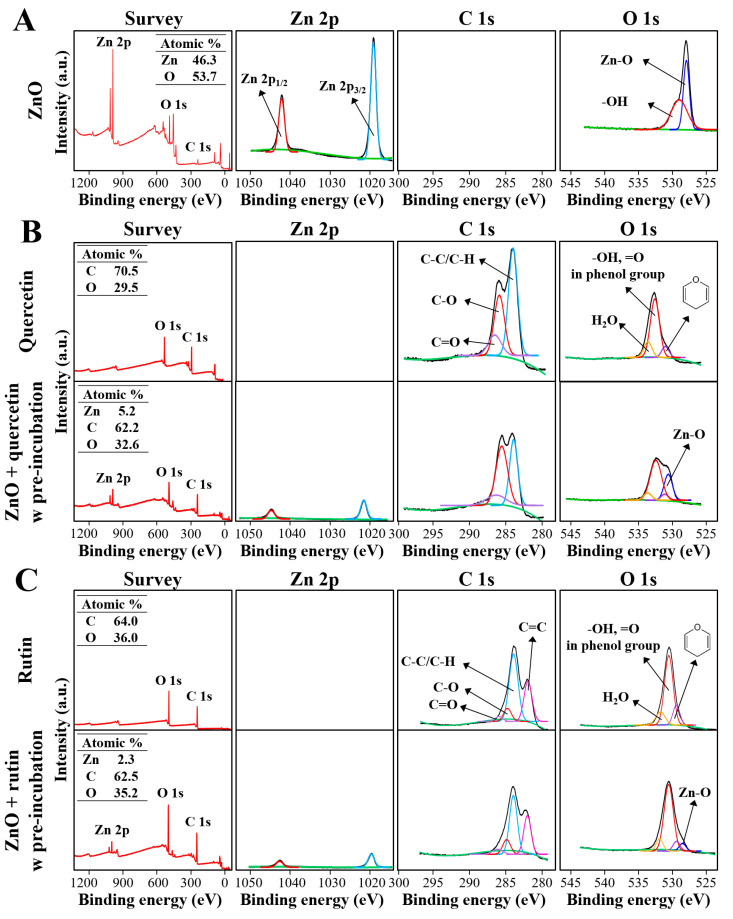
(**A**) X-ray photoelectron spectroscopy (XPS) survey and high-resolution spectra of pristine ZnO NPs, (**B**) quercetin only and quercetin pre-incubated with ZnO NPs, and (**C**) rutin only and rutin pre-incubated with ZnO NPs.

The characterization on the interactions was further carried out by XPS analysis after pre-incubation, which provides information about elemental composition and chemical state. The spectra survey shows that the atomic Zn level (46.3%) of ZnO NPs dramatically decreased to 5.2% and 2.3% after pre-incubation with quercetin and rutin, respectively (Figure 7). The C level (70.5%) of quercetin also decreased to 62.2% after pre-incubation with ZnO NPs, whereas no remarkable change in the C level of rutin was found, indicating stronger formation of ZnO-quercetin corona than ZnO-rutin corona. The O levels of quercetin and rutin remained almost constant, but a decreased O level (53.7% to 32.6–35.2%) of ZnO was found after pre-incubation with quercetin or rutin. These results suggest that the surface of ZnO NPs was coated by quercetin or rutin to some extent, resulting in the quenching of the exposure of Zn and O elements to the environment.

The spectrum of each element shows that the Zn 2p core-levels of ZnO NPs at 1042.8 eV and 1019.8 eV attributed to Zn 2p_1/2_ and Zn 2p_3/2_ [44] (Figure 7A), respectively, significantly decreased by interactions with two polyphenols (Figure 7B,C). This result is in good agreement with the elemental composition (Figure 7) and FTIR (Figure 6) results. The O 1s spectrum of ZnO shows two different forms of oxygen (Figure 7A); the binding energy peaks at 529.6 eV and 528.5 eV are assigned to OH group absorbed onto the surface of ZnO NPs in the atmosphere [44,45] and O^2−^ ions in the Zn–O bonding of the wurtzite structure of ZnO [44], respectively. The peak at 528.5 eV attributed to O^2−^ ions in the Zn–O remarkably decreased by interaction with quercetin or rutin (Figure 7B,C) in a similar manner to Zn 2p.

In the case of quercetin, the C 1s spectrum shows peaks at 287.1 eV, 285.6 eV, and 284.6 eV (Figure 7B), attributed to C=O, C–O, and C–C/C–H surrounding groups [46,47]. Among them, significantly decreased intensity of C=O and C–C/C–H groups was found by interaction with ZnO NPs. The O 1s spectrum of pure quercetin was deconvoluted into three peaks attributed to H_2_O in the commercial quercetin, –OH and =O in the phenol group, and the oxygen of the pyran structure at 534.2 eV, 533.2 eV, and 531.9 eV, respectively. However, the intensity of all the O 1s peaks decreased by interaction with ZnO NPs. Decreased and slightly shifted Zn-O peak at 531.7 eV was also found, indicating the interactions between ZnO NPs and quercetin.

The C 1s spectrum of pure rutin was deconvoluted into four peaks at 286.6 eV, 285.4 eV, 284.6 eV, and 282.7 eV, assigned to C=O, C–O, C–C/C–H, and C=O binding energies, respectively (Figure 7C) [48,49]. The O 1s spectrum of rutin shows the same peaks at 534.2 eV, 533.2 eV, and 531.9 eV as observed in the O 1s spectrum of quercetin, corresponding to H_2_O in rutin trihydrate, –OH and =O in the phenol group, and the oxygen of the pyran structure, respectively. Decreased and slightly shifted Zn-O peak at 531.0 eV was also present. However, the C 1s and O 1s spectra of rutin were not remarkably affected by the interaction with ZnO NPs. All XPS results clearly demonstrate the interactions between ZnO NPs and quercetin or rutin. The chemical compositions and surface chemical states were more affected by ZnO interaction with quercetin than ZnO with rutin. Based on Figure 6 and Figure 7, the changes in chemical characteristics, elemental composition, and chemical sate on the surface of ZnO-quercetin corona were more remarkable than ZnO-rutin corona, which seems to induce high cytotoxicity (Figure 2) and ex vivo intestinal absorption (Figure 4) of ZnO NPs. Meanwhile, relatively strong formation of ZnO-quercetin corona may protect ZnO dissolution under neutral pH conditions such as saliva and intestinal fluids compared with ZnO-rutin corona (Figure 5). It is worth noting that the crystallinity of ZnO NPs and chemical bondings of ZnO NPs, quercetin, or rutin did not change, although the degree of exposure of surface chemical bondings were quenched to some extent. This result suggests that the interactions may occur by electrostatic interactions or Van der Waals forces. The mechanism of action for the interactions is required to be elucidated.

## 4. Conclusions

The interactions between ZnO NPs and representative two polyphenols (quercetin and rutin) were investigated in terms of cytotoxicity, ABTS and DPPH radical scavenging activities, ex vivo intestinal absorption, and ZnO solubility. Moreover, the interactions were characterized by analyzing the changes in particle size, crystallinity, surface chemical bonding intensity, chemical composition, and surface chemical state. The results demonstrate that the interactions caused high cell proliferation inhibition, LDH release, ROS generation, and ex vivo intestinal absorption of ZnO NPs, especially after pre-incubation with quercetin compared with rutin, whereas ABTS and DPPH radical scavenging activities and ex vivo intestinal transports of two polyphenols were not affected by the interactions. Physico-chemical and surface characterization revealed that the interactions did not affect the crystallinity of ZnO NPs, but the hydrodynamic diameters of ZnO NPs were reduced, and the degree of exposure of surface chemical bondings of ZnO, quercetin, or rutin were quenched to some extent by the interactions. Elemental compositions and surface chemical group intensities also decreased by the interactions, especially by ZnO NP interaction with quercetin. It is, therefore, concluded that the interactions affect chemical characteristics and surface chemical states of ZnO NPs, quercetin, or rutin, which can also influence cytotoxicity, intestinal absorption, and solubility of ZnO NPs. Relatively strong ZnO NP interaction with quercetin can cause higher cytotoxicity and ex vivo intestinal transport than ZnO NP interacted with rutin or pristine ZnO NPs, requiring further investigation on in vivo experiments, characterization, and mechanism of action.

## Figures and Tables

**Table 1:** Hydrodynamic diameters and zeta potentials of pristine ZnO NPs and ZnO NP in the presence of quercetin or rutin without or with pre-incubation.

Samples	DW		MEM		Tyrode’s Solution		Digestion Fluids	
	Hydrodynamic Diameters (nm)	Zeta Potential (mV)	Hydrodynamic Diameters (nm)	Zeta Potential (mV)	Hydrodynamic Diameters (nm)	Zeta Potential (mV)	Hydrodynamic Diameters (nm)	Zeta Potential (mV)
ZnO	346 ± 9 ^a^	18.5 ± 0.9 ^a^	330 ± 3 ^a^	−9.7 ± 0.4 ^a^	564 ± 52 ^a^	−9.4 ± 0.1 ^a^	5811 ± 470 ^a^	−23.7 ± 0.3 ^a^
ZnO + quercetin *w/o* pre-incubation	333 ± 29 ^a^	−15.2 ± 0.4 ^b^	337 ± 8 ^a^	−9.3 ± 0.4 ^a^	541 ± 11 ^a^	−15.7 ± 1.3 ^b^	5448 ± 351 ^a^	−23.2 ± 0.9 ^a^
ZnO + quercetin w pre-incubation	270 ± 7 ^b^	−14.8 ± 0.1 ^b^	306 ± 9 ^b^	−9.6 ± 0.2 ^a^	346 ± 28 ^c^	−15.7 ± 0.6 ^b^	2489 ± 273 ^c^	−23.5 ± 1.3 ^a^
ZnO + rutin *w/o* pre-incubation	232 ± 4 ^b^	−16.6 ± 0.4 ^c^	297 ± 3 ^b^	−9.4 ± 0.2 ^a^	562 ± 29 ^a^	−10.7 ± 0.5 ^a^	5379 ± 286 ^a^	−23.3 ± 0.9 ^a^
ZnO + rutin w pre-incubation	212 ± 3 ^c^	−17.3 ± 0.4 ^c^	279 ± 5 ^c^	−9.8 ± 0.6 ^a^	449 ± 9 ^b^	−9.9 ± 0.5 ^a^	3386 ± 244 ^b^	−25.3 ± 0.7 ^a^

Different lower-case letters (a,b,c) indicate significant differences among pristine ZnO and ZnO in the presence of quercetin or rutin without (*w*/*o*) or with (*w*) pre-incubation (*p* < 0.05). Abbreviations: DW, distilled water; MEM, minimum essential medium.

## Data Availability

The data presented in this study are available in the article and Supplementary Materials.

## Supplementary Materials

The following supporting information can be downloaded at: https://www.mdpi.com/article/10.3390/nano12193337/s1, Figure S1: Scanning electron microscopic (SEM) images and size distribution of ZnO NPs; Table S1: Compositions of in vitro three-steps of digestion fluids.
