# Supplementary material for: Interactions between ZnO Nanoparticles and Polyphenols Affect Biological Responses
Source: Nanomaterials (Basel). 2022 Sep 25;12(19):3337. doi: 10.3390/nano12193337 (PMC9565856; doi:10.3390/nano12193337)
Supplement: Supplementary file 1 [file nanomaterials-12-03337-s001.zip › nanomaterials-1913220-supplementary.pdf]

nanomaterials

Supplementary Materials

Interactions between ZnO Nanoparticles and Polyphenols Affect Biological Responses

Su-Bin Kim, Na-Kyung Yoo and Soo-Jin Choi *

Division of Applied Food System, Major of Food Science & Technology, Seoul Women's University, Seoul 01797, Korea
* Correspondence: sjchoi@swu.ac.kr; Tel.: +82-2-970-5634

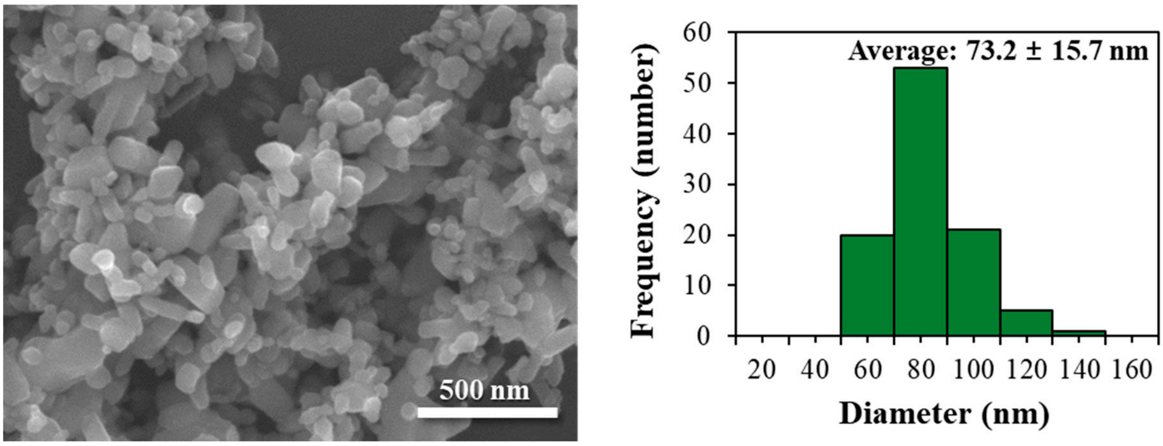

Figure S1. Scanning electron microscopy (SEM) images and size distributions of ZnO NPs. Particle size distributions were determined by randomly selecting 100 particles from SEM images.

Table S1. Composition of simulated digestion fluids of the in vitro digestion model.

Digestion fluids	pH	Composition (amounts based on 1 L of digestion juices)
**Saliva**	6.8±0.1	KCl (896 mg), KSCN (200 mg), $NaH_2PO_4 \cdot 2H_2O$ (1110.3 mg), Na_2SO_4 (570 mg), NaCl (298 mg), $NaHCO_3$ (1694 mg), urea (200 mg), α-amylase (290 mg), uric acid (15 mg), mucin (25 mg)
**Gastric juice**	1.3±0.1	NaCl (2752 mg) , $NaH_2PO_4 \cdot 2H_2O$ (332.8 mg), KCl (824 mg), $CaCl_2 \cdot 2H_2O$ (400 mg), urea (85 mg), D-(+)-glucose (650 mg), glucuronic acid (20 mg), glucosamine hydrochloride (330 mg), BSA (1 g), pepsin (2.5 g), mucin (3 g)
**Duodenal juice**	8.1±0.1	NaCl (7012 mg), $NaHCO_3$ (3388 mg), KH_2PO_4 (80 mg), KCl (564 mg), $MgCl_2$ (22.7 mg), 37% HCl (180 µL), urea (100 mg), BSA (1 g), pancreatin (9 g), lipase (1.5 g), $CaCl_2 \cdot 2H_2O$ (200 mg)
**Bile juice**	8.2±0.1	NaCl (5259 mg), $NaHCO_3$ (5785 mg), KCl (376 mg), 37% HCl (150 µL), urea (250 mg), BSA (1.8 g), bile (30 g), $CaCl_2 \cdot 2H_2O$ (222 mg)
